# Innate immune responses in acute HIV-1 infection: protective or pathogenic?

**DOI:** 10.1186/1742-4690-10-S1-O33

**Published:** 2013-09-19

**Authors:** Persephone Borrow, Andrea Stacey, Angharad Fenton-May, Oliver Dibben, Elizabeth Haygreen, Tanja Emmerich, Norma Kim, Elizabeth Marshall, Kerry Lavender, Myron Cohen, Paul Goepfert, Ian Williams, Dennis Wallace, George Shaw, Beatrice Hahn, Christina Ochsenbauer, John Kappes, Philip Norris, Andrew McMichael, Barton Haynes

**Affiliations:** 1 Nuffield Department of Clinical Medicine, University of Oxford, Oxford, UK; 2 RTI International, Durham, NC, USA; 3 Departments of Microbiology and Immunology, University of North Carolina at Chapel Hill, Chapel Hill, NC, USA; 4 Department of Medicine, University of Alabama at Birmingham, Birmingham, AL, USA; 5 Center for Sexual Health and HIV Research, University College London, London, UK; 6 Department of Microbiology, Perelman School of Medicine, University of Pennsylvania, Philadelphia, PA, USA; 7 Blood Systems Research Institute, San Francisco, CA, USA; 8 Department of Medicine, Duke University, Durham, NC, USA

## Background

The importance of events in acute HIV-1 infection (AHI) in determining the subsequent disease course prompts a need to understand the virus-immune system interactions in this phase of infection that impact on concurrent and ensuing viral replication and pathogenesis. The speed with which innate responses can be mobilized following pathogen exposure suggests they may play critical roles in AHI. We sought to characterise the innate responses activated during AHI and identify components of the response that contribute to control of viremia or conversely promote immune activation and virus replication.

## Materials and methods

Peripheral blood samples were cryopreserved at serial timepoints from subjects acutely infected with HIV. The dynamics of systemic up-regulation of cytokines/chemokines were analysed using Luminex multiplex assays and ELISAs, and activation of innate cell subsets was evaluated by multiparameter flow cytometry. Spearman correlation analyses and multiple regression modelling were employed to determine relationships between innate variables and assess their impact on viral control.

Primary HIV isolates were derived from plasma cryopreserved at timepoints in acute and chronic infection and their sensitivity to control by IFN alpha and IFN beta was evaluated using *in vitro* assays. The IFN sensitivity of viruses generated from infectious molecular clones (IMCs) corresponding to deduced founder and paired 6-month virus sequences was also assessed.

## Results

The increase in viremia in AHI was associated with rapid activation of systemic innate responses, evidenced by a reduction in the circulating dendritic cell frequency, elevations in plasma levels of type 1 interferon (IFN) and other cytokines and chemokines, and natural killer (NK) cell activation and proliferation. Some components of the innate response, e.g. strong NK cell responses, correlated with establishment of lower set-point viral loads, whereas others, such as inflammatory cytokine production, correlated with establishment of higher set-point viral loads. However multiple regression modelling illustrated that the association between any one component of the innate response and set-point viremia was confounded by other interlinked innate variables.

To seek evidence that type 1 IFN exerts selective pressure on HIV replication during the establishment of infection, the IFN-resistance of HIV isolates derived from AHI subjects and founder virus IMCs was compared to that of virus isolates/IMCs generated from the same subjects later in infection. Founder viruses were found to be significantly more IFN-resistant than matched viruses present in the same subjects at later times post-infection, suggesting that type I IFN plays an important role in HIV control during the initial stages of infection and may drive selection for IFN-resistant founder viruses from the transmitted virus pool.

## Conclusions

Identification of type 1 IFN-mediated antiviral activity and NK effector activity as mechanisms that contribute to HIV-1 control during the initial stages of infection could enable harnessing of these activities to complement the protection afforded by vaccine-elicited adaptive responses.

